# (2*E*)-1-(2-Bromo­phen­yl)-3-(4-bromo­phen­yl)prop-2-en-1-one

**DOI:** 10.1107/S1600536810022956

**Published:** 2010-06-18

**Authors:** Jerry P. Jasinski, Ray J. Butcher, K. Veena, B. Narayana, H. S. Yathirajan

**Affiliations:** a Department of Chemistry, Keene State College, 229 Main Street, Keene, NH 03435-2001, USA; b Department of Chemistry, Howard University, 525 College Street NW, Washington, DC 20059, USA; c Department of Studies in Chemistry, Mangalore University, Mangalagangotri 574 199, India; d Department of Studies in Chemistry, University of Mysore, Manasagangotri, Mysore 570 006, India

## Abstract

The title compound, C_15_H_10_Br_2_O, is a chalcone with 2-bromo­phenyl and 4-bromo­phenyl rings bonded to opposite sides of a propenone group. The dihedral angle between mean planes of the benzene rings is 71.3 (1)°. The angle between the mean plane of the prop-2-ene-1-one group and the mean planes of the 2-bromo­phenyl and 4-bromo­phenyl rings are 64.2 (9) and 71.3 (1)°, respectively. A weak inter­molecular C—H⋯O inter­action and two weak C—Br⋯π inter­actions are observed, which contribute to the stability of the crystal packing.

## Related literature

For the radical quenching properties of included phenol groups, see: Dhar (1981 ▶). For the biological activity of chalcones, see: Dimmock *et al.* (1999 ▶). For related structures, see: Ng *et al.* (2006 ▶); Teh *et al.* (2006 ▶). For bond-length data, see: Allen *et al.* (1987 ▶)
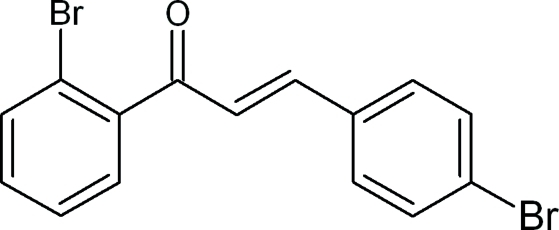


## Experimental

### 

#### Crystal data

C_15_H_10_Br_2_O
*M* *_r_* = 366.05
Monoclinic, 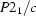

*a* = 5.6988 (5) Å
*b* = 9.5462 (9) Å
*c* = 23.8532 (15) Å
β = 91.021 (8)°
*V* = 1297.46 (18) Å^3^
*Z* = 4
Cu *K*α radiation
μ = 7.79 mm^−1^
*T* = 110 K
0.62 × 0.47 × 0.26 mm

#### Data collection

Oxford Diffraction Xcalibur Ruby Gemini diffractometer
Absorption correction: analytical (*CrysAlis RED*; Oxford Diffraction, 2007 ▶) *T*_min_ = 0.078, *T*_max_ = 0.315
4592 measured reflections
2532 independent reflections
2454 reflections with *I* > 2σ(*I*)
*R*_int_ = 0.027

#### Refinement

*R*[*F* ^2^ > 2σ(*F* ^2^)] = 0.045
*wR*(*F* ^2^) = 0.152
*S* = 1.32
2532 reflections
164 parameters
H-atom parameters constrained
Δρ_max_ = 1.27 e Å^−3^
Δρ_min_ = −1.00 e Å^−3^

### 

Data collection: *CrysAlis PRO* (Oxford Diffraction, 2007 ▶); cell refinement: *CrysAlis PRO*; data reduction: *CrysAlis PRO*; program(s) used to solve structure: *SHELXS97* (Sheldrick, 2008 ▶); program(s) used to refine structure: *SHELXL97* (Sheldrick, 2008 ▶); molecular graphics: *SHELXTL* (Sheldrick, 2008 ▶); software used to prepare material for publication: *SHELXTL*.

## Supplementary Material

Crystal structure: contains datablocks global, I. DOI: 10.1107/S1600536810022956/dn2577sup1.cif

Structure factors: contains datablocks I. DOI: 10.1107/S1600536810022956/dn2577Isup2.hkl

Additional supplementary materials:  crystallographic information; 3D view; checkCIF report

## Figures and Tables

**Table 1:** Hydrogen-bond geometry (Å, °)

*D*—H⋯*A*	*D*—H	H⋯*A*	*D*⋯*A*	*D*—H⋯*A*
C12—H12*A*⋯O1^i^	0.95	2.46	3.368 (7)	159

Symmetry code: (i) 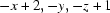.

**Table 2:** C—Br⋯π inter­actions (Å, °) *Cg*1 and *Cg*2 are the centroids of the C1–C6 and C10–C15 rings, respectively.

	Br1⋯*Cg*2	Br1–Perp	C2—Br1⋯*Cg*2
C2—Br1⋯*Cg*2^i^	3.522 (2)	3.488	154.82 (17)
C13—-Br2⋯*Cg*1^ii^	3.827 (2)	3.377	165.44 (17)

Symmetry codes: (i) 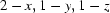; (ii) 
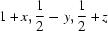
.

**Fig. 1.**
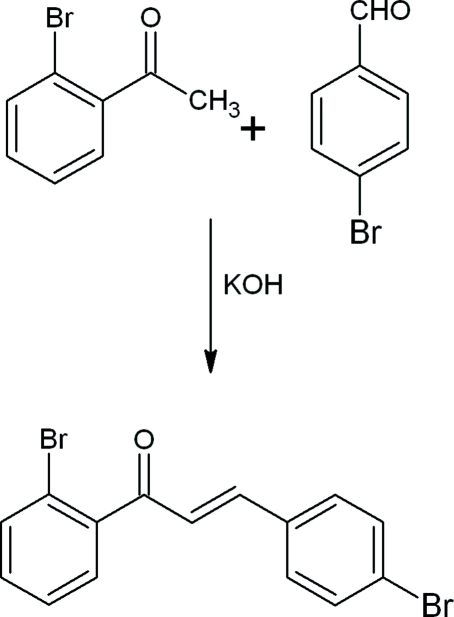
Reaction Scheme for the title compound.

**Fig. 2.**
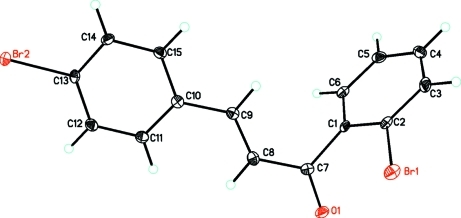
Molecular structure of the title compound, C15H10Br2O, showing the atom labeling scheme and 50% probability displacement ellipsoids.

## supplementary crystallographic information

### Comment

Chalcones, or 1,3-diaryl-2-propen-1-ones, belong to the flavonoid family. Chemically they consist of open-chain flavonoids in which the two aromatic rings are joined by a three-carbon α,β-unsaturated carbonyl system. A vast number of naturally occurring chalcones are polyhydroxylated in the aryl rings. The radical quenching properties of the phenol groups present in many chalcones have raised interest in using the compounds or chalcone rich plant extracts as drugs or food preservatives (Dhar, 1981). Chalcones have been reported to possess many useful properties, including anti-inflammatory, antimicrobial, antifungal, antioxidant, cytotoxic, anticancer activities (Dimmock *et al.*, 1999). The crystal structures of closely related chalcones, *viz*., 1,3-bis(4-bromophenyl)prop-2-en-1-one (Ng *et al.*, 2006) and 3-(3-bromophenyl)-1-(4-bromophenyl)prop-2-en-1-one (Teh *et al.*, 2006) have been reported. Hence in continuation with the synthesis and crystal structure determination and also owing to the importance of these flavanoid analogs, this bromo chalcone, C_15_H_10_Br_2_O, is synthesized and its crystal structure is reported.

The title compound, C_15_H_10_Br_2_O, is a chalcone with 2-bromophenyl and 4-bromophenyl rings bonded to opposite sides of a propenone group (Fig. 2). The dihedral angle between mean planes of the benzene rings in the *ortho*-bromo and *para*-bromo substituted rings is 71.3 (1)°. The angle between the mean plane of the prop-2-ene-1-one group (C1/C7/O1/C8) and the mean planes of the benzene rings in the 2-bromophenyl (C1–C6) and 4-bromophenyl rings (C10–C15) are 64.2 (9)° and 71.3 (1)°, respectively. Bond distances and angles are in normal ranges (Allen *et al.*, 1987). While no classical hydrogen bonds are present, a weak intermolecular C12—H12A···O1 interaction (Table 1) and two weak π-ring intermolecular interactions (Table 2) are observed which contribute to the stability of crystal packing.

### Experimental

A 50% KOH solution was added to a mixture of 2-bromo acetophenone (0.01 mol, 1.99 g) and 4-bromo benzaldehyde (0.01 mol, 1.85 g) in 25 ml of ethanol (Fig. 1). The mixture was stirred for an hour at room temperature and the precipitate was collected by filtration and purified by recrystallization from ethanol. The single-crystal was grown from ethyl acetate by slow evaporation method and yield of the compound was 68% (m.p.373–375 K). Analytical data: Found (Calculated): C %: 49.19 (49.22%); H%: 2.73 (2.75%).

### Refinement

The H atoms were placed in geometrically idealized positions and constrained to ride on their parent atoms with C–H distances = 0.95Å and with *U*_iso_(H) = 1.17–1.22 *U*_eq_(C).

### Crystal data

**Table:**

C_15_H_10_Br_2_O	*F*(000) = 712
*M**_r_* = 366.05	*D*_x_ = 1.874 Mg m^−^^3^
Monoclinic, *P*2_1_/*c*	Cu *K*α radiation, λ = 1.54178 Å
Hall symbol: -P 2ybc	Cell parameters from 3417 reflections
*a* = 5.6988 (5) Å	θ = 4.6–74.1°
*b* = 9.5462 (9) Å	µ = 7.79 mm^−^^1^
*c* = 23.8532 (15) Å	*T* = 110 K
β = 91.021 (8)°	Prism, colorless
*V* = 1297.46 (18) Å^3^	0.62 × 0.47 × 0.26 mm
*Z* = 4	

### Data collection

**Table:**

Oxford Diffraction Xcalibur Ruby Gemini diffractometer	2532 independent reflections
Radiation source: Enhance (Cu) X-ray Source	2454 reflections with *I* > 2σ(*I*)
graphite	*R*_int_ = 0.027
Detector resolution: 10.5081 pixels mm^-1^	θ_max_ = 74.1°, θ_min_ = 5.0°
ω scans	*h* = −6→6
Absorption correction: analytical (*CrysAlis RED*; Oxford Diffraction, 2007)	*k* = −6→11
*T*_min_ = 0.078, *T*_max_ = 0.315	*l* = −29→25
4592 measured reflections	

### Refinement

**Table:**

Refinement on *F*^2^	Secondary atom site location: difference Fourier map
Least-squares matrix: full	Hydrogen site location: inferred from neighbouring sites
*R*[*F*^2^ > 2σ(*F*^2^)] = 0.045	H-atom parameters constrained
*wR*(*F*^2^) = 0.152	*w* = 1/[σ^2^(*F*_o_^2^) + (0.0631*P*)^2^ + 9.323*P*] where *P* = (*F*_o_^2^ + 2*F*_c_^2^)/3
*S* = 1.32	(Δ/σ)_max_ < 0.001
2532 reflections	Δρ_max_ = 1.27 e Å^−^^3^
164 parameters	Δρ_min_ = −1.00 e Å^−^^3^
0 restraints	Extinction correction: *SHELXL97* (Sheldrick, 2008), Fc^*^=kFc[1+0.001xFc^2^λ^3^/sin(2θ)]^-1/4^
Primary atom site location: structure-invariant direct methods	Extinction coefficient: 0.0029 (4)

### Special details

**Table:**

Experimental. IR data (KBr) ν cm^-1^: 3048 cm^-1^ (C—H str) 1671 cm^-1^ (C=O), 1685 cm^-1^ (C=C).
Geometry. All e.s.d.'s (except the e.s.d. in the dihedral angle between two l.s. planes) are estimated using the full covariance matrix. The cell e.s.d.'s are taken into account individually in the estimation of e.s.d.'s in distances, angles and torsion angles; correlations between e.s.d.'s in cell parameters are only used when they are defined by crystal symmetry. An approximate (isotropic) treatment of cell e.s.d.'s is used for estimating e.s.d.'s involving l.s. planes.
Refinement. Refinement of *F*^2^ against ALL reflections. The weighted *R*-factor *wR* and goodness of fit *S* are based on *F*^2^, conventional *R*-factors *R* are based on *F*, with *F* set to zero for negative *F*^2^. The threshold expression of *F*^2^ > σ(*F*^2^) is used only for calculating *R*-factors(gt) *etc*. and is not relevant to the choice of reflections for refinement. *R*-factors based on *F*^2^ are statistically about twice as large as those based on *F*, and *R*- factors based on ALL data will be even larger.

### Fractional atomic coordinates and isotropic or equivalent isotropic displacement parameters (Å^2^)

**Table:**

	*x*	*y*	*z*	*U*_iso_*/*U*_eq_	
Br1	1.27853 (11)	0.55820 (6)	0.36129 (3)	0.0217 (3)	
Br2	0.14170 (10)	0.10866 (6)	0.65074 (2)	0.0182 (2)	
O1	1.2468 (7)	0.2116 (5)	0.37198 (18)	0.0211 (9)	
C1	0.9316 (10)	0.3480 (6)	0.3361 (2)	0.0144 (11)	
C2	1.0220 (10)	0.4768 (6)	0.3200 (2)	0.0157 (11)	
C3	0.9269 (12)	0.5525 (7)	0.2759 (3)	0.0223 (13)	
H3A	0.9912	0.6406	0.2658	0.027*	
C4	0.7337 (12)	0.4969 (7)	0.2462 (2)	0.0239 (14)	
H4A	0.6670	0.5468	0.2154	0.029*	
C5	0.6406 (11)	0.3698 (7)	0.2619 (3)	0.0219 (13)	
H5A	0.5103	0.3320	0.2416	0.026*	
C6	0.7359 (10)	0.2973 (6)	0.3069 (2)	0.0180 (12)	
H6A	0.6669	0.2114	0.3180	0.022*	
C7	1.0493 (10)	0.2574 (6)	0.3798 (2)	0.0149 (11)	
C8	0.9223 (11)	0.2193 (6)	0.4304 (2)	0.0181 (12)	
H8A	0.9888	0.1485	0.4537	0.022*	
C9	0.7192 (10)	0.2767 (6)	0.4462 (2)	0.0162 (11)	
H9A	0.6527	0.3466	0.4225	0.019*	
C10	0.5903 (10)	0.2408 (6)	0.4972 (2)	0.0162 (11)	
C11	0.6596 (11)	0.1294 (7)	0.5320 (3)	0.0201 (12)	
H11A	0.7986	0.0790	0.5238	0.024*	
C12	0.5304 (11)	0.0913 (6)	0.5780 (3)	0.0190 (12)	
H12A	0.5788	0.0155	0.6013	0.023*	
C13	0.3279 (10)	0.1664 (6)	0.5895 (2)	0.0151 (11)	
C14	0.2554 (10)	0.2787 (6)	0.5566 (2)	0.0180 (12)	
H14A	0.1178	0.3299	0.5654	0.022*	
C15	0.3891 (10)	0.3146 (6)	0.5105 (2)	0.0173 (12)	
H15A	0.3415	0.3914	0.4877	0.021*	

### Atomic displacement parameters (Å^2^)

**Table:**

	*U*^11^	*U*^22^	*U*^33^	*U*^12^	*U*^13^	*U*^23^
Br1	0.0195 (4)	0.0157 (4)	0.0300 (4)	−0.0029 (2)	0.0034 (3)	−0.0038 (2)
Br2	0.0190 (4)	0.0196 (4)	0.0161 (3)	−0.0020 (2)	0.0042 (2)	0.0011 (2)
O1	0.015 (2)	0.021 (2)	0.027 (2)	0.0034 (17)	0.0043 (16)	0.0015 (18)
C1	0.015 (3)	0.016 (3)	0.012 (2)	0.004 (2)	0.007 (2)	−0.001 (2)
C2	0.013 (3)	0.017 (3)	0.017 (3)	−0.001 (2)	0.005 (2)	−0.003 (2)
C3	0.028 (3)	0.020 (3)	0.019 (3)	0.006 (2)	0.012 (2)	0.002 (2)
C4	0.027 (3)	0.030 (4)	0.015 (3)	0.014 (3)	0.004 (2)	0.003 (2)
C5	0.018 (3)	0.029 (3)	0.019 (3)	0.007 (2)	0.001 (2)	−0.005 (2)
C6	0.013 (3)	0.019 (3)	0.022 (3)	−0.001 (2)	0.004 (2)	−0.001 (2)
C7	0.015 (3)	0.009 (2)	0.020 (3)	−0.001 (2)	−0.001 (2)	−0.004 (2)
C8	0.023 (3)	0.014 (3)	0.017 (3)	−0.002 (2)	0.000 (2)	0.000 (2)
C9	0.017 (3)	0.013 (3)	0.018 (3)	−0.001 (2)	−0.002 (2)	0.000 (2)
C10	0.018 (3)	0.014 (3)	0.017 (3)	−0.002 (2)	−0.001 (2)	−0.001 (2)
C11	0.020 (3)	0.018 (3)	0.022 (3)	0.005 (2)	0.001 (2)	0.002 (2)
C12	0.022 (3)	0.016 (3)	0.019 (3)	0.003 (2)	0.000 (2)	0.003 (2)
C13	0.018 (3)	0.016 (3)	0.011 (2)	−0.003 (2)	0.002 (2)	−0.001 (2)
C14	0.014 (3)	0.020 (3)	0.020 (3)	0.003 (2)	0.002 (2)	−0.001 (2)
C15	0.017 (3)	0.016 (3)	0.020 (3)	0.000 (2)	−0.001 (2)	0.003 (2)

### Geometric parameters (Å, °)

**Table:**

Br1—C2	1.913 (6)	C8—C9	1.341 (9)
Br2—C13	1.903 (6)	C8—H8A	0.9500
O1—C7	1.225 (7)	C9—C10	1.471 (8)
C1—C2	1.389 (8)	C9—H9A	0.9500
C1—C6	1.391 (8)	C10—C15	1.388 (8)
C1—C7	1.504 (8)	C10—C11	1.402 (8)
C2—C3	1.380 (9)	C11—C12	1.381 (9)
C3—C4	1.403 (10)	C11—H11A	0.9500
C3—H3A	0.9500	C12—C13	1.390 (9)
C4—C5	1.378 (10)	C12—H12A	0.9500
C4—H4A	0.9500	C13—C14	1.386 (8)
C5—C6	1.380 (9)	C14—C15	1.392 (8)
C5—H5A	0.9500	C14—H14A	0.9500
C6—H6A	0.9500	C15—H15A	0.9500
C7—C8	1.463 (8)		
			
C2—C1—C6	117.9 (5)	C7—C8—H8A	117.5
C2—C1—C7	122.5 (5)	C8—C9—C10	125.7 (5)
C6—C1—C7	119.4 (5)	C8—C9—H9A	117.1
C3—C2—C1	122.1 (6)	C10—C9—H9A	117.1
C3—C2—Br1	117.8 (5)	C15—C10—C11	118.3 (5)
C1—C2—Br1	120.1 (4)	C15—C10—C9	119.9 (5)
C2—C3—C4	118.7 (6)	C11—C10—C9	121.8 (5)
C2—C3—H3A	120.6	C12—C11—C10	121.5 (6)
C4—C3—H3A	120.6	C12—C11—H11A	119.3
C5—C4—C3	119.9 (6)	C10—C11—H11A	119.3
C5—C4—H4A	120.0	C11—C12—C13	118.4 (5)
C3—C4—H4A	120.0	C11—C12—H12A	120.8
C4—C5—C6	120.3 (6)	C13—C12—H12A	120.8
C4—C5—H5A	119.9	C14—C13—C12	121.9 (5)
C6—C5—H5A	119.9	C14—C13—Br2	119.6 (4)
C5—C6—C1	121.1 (6)	C12—C13—Br2	118.5 (4)
C5—C6—H6A	119.5	C13—C14—C15	118.4 (5)
C1—C6—H6A	119.5	C13—C14—H14A	120.8
O1—C7—C8	120.4 (5)	C15—C14—H14A	120.8
O1—C7—C1	120.0 (5)	C10—C15—C14	121.5 (5)
C8—C7—C1	119.6 (5)	C10—C15—H15A	119.3
C9—C8—C7	124.9 (5)	C14—C15—H15A	119.3
C9—C8—H8A	117.5		
			
C6—C1—C2—C3	−1.4 (8)	O1—C7—C8—C9	171.3 (6)
C7—C1—C2—C3	173.0 (5)	C1—C7—C8—C9	−11.4 (9)
C6—C1—C2—Br1	176.2 (4)	C7—C8—C9—C10	−179.3 (5)
C7—C1—C2—Br1	−9.4 (7)	C8—C9—C10—C15	175.9 (6)
C1—C2—C3—C4	−0.3 (9)	C8—C9—C10—C11	−6.7 (9)
Br1—C2—C3—C4	−177.9 (4)	C15—C10—C11—C12	1.2 (9)
C2—C3—C4—C5	0.8 (9)	C9—C10—C11—C12	−176.3 (6)
C3—C4—C5—C6	0.4 (9)	C10—C11—C12—C13	−0.2 (9)
C4—C5—C6—C1	−2.1 (9)	C11—C12—C13—C14	−0.9 (9)
C2—C1—C6—C5	2.6 (8)	C11—C12—C13—Br2	177.1 (5)
C7—C1—C6—C5	−172.0 (5)	C12—C13—C14—C15	1.0 (9)
C2—C1—C7—O1	−62.2 (7)	Br2—C13—C14—C15	−177.1 (4)
C6—C1—C7—O1	112.2 (6)	C11—C10—C15—C14	−1.1 (9)
C2—C1—C7—C8	120.6 (6)	C9—C10—C15—C14	176.4 (5)
C6—C1—C7—C8	−65.1 (7)	C13—C14—C15—C10	0.1 (9)

### Hydrogen-bond geometry (Å, °)

**Table:**

*D*—H···*A*	*D*—H	H···*A*	*D*···*A*	*D*—H···*A*
C12—H12A···O1^i^	0.95	2.46	3.368 (7)	159

Symmetry codes: (i) −*x*+2, −*y*, −*z*+1.

### Table 2 C—Br···π interactions (Å, °)

Cg1 and Cg2 are the centroids of the C1–C6 and C10–C15 rings, respectively.

**Table:**

	Br1···Cg2	Br1–Perp	C2—Br1···Cg2
C2—Br1···Cg2^i^	3.522 (2)	3.488	154.82 (17)
C13—-Br2···Cg1^ii^	3.827 (2)	3.377	165.44 (17)

Symmetry codes: (i) 2-x, 1-y, 1-z; (ii) 1+x, 1/2-y, 1/2+z.
